# Complete Genome Sequence of an Avian Paramyxovirus Type 4 from North America Reveals a Shorter Genome and New Genotype

**DOI:** 10.1128/genomeA.00075-12

**Published:** 2013-01-31

**Authors:** Manoharan Parthiban, Manimaran Kaliyaperumal, Sa Xiao, Baibaswata Nayak, Anandan Paldurai, Shin-Hee Kim, Brian S. Ladman, Lauren A. Preskenis, Jack Gelb, Peter L. Collins, Siba K. Samal

**Affiliations:** a Virginia-Maryland Regional College of Veterinary Medicine, University of Maryland, College Park, Maryland, USA; b Department of Animal and Food Sciences, University of Delaware, Newark, Delaware, USA; c Laboratory of Infectious Diseases, National Institute of Allergy and Infectious Diseases, National Institutes of Health, Bethesda, Maryland, USA

## Abstract

An avian paramyxovirus type 4 (APMV-4) was isolated from a duck in Delaware in 2010. Its genome is 15,048 nucleotides (nt) long, which is shorter by 6 nt than those for all previously reported strains. Phylogenetic analysis revealed that this strain formed a separate cluster within APMV-4 strains.

## GENOME ANNOUNCEMENT

Avian paramyxoviruses (APMVs) belong to the genus *Avulavirus* in the family *Paramyxoviridae* and include at least nine serotypes, APMV-1 to APMV-9 (1, 2). APMV-1, or Newcastle disease virus (NDV), is the most extensively studied APMV (1–3). APMV-4 is frequently isolated from waterfowl around the world (1–4). To date, complete genome sequences of APMV-4 strains are available for strains that were isolated from ducks in Hong Kong, Korea, Belgium, and South Africa over a period of 37 years (5–8). In this study, we report the complete genome sequence of an isolate of APMV-4 in North America.

APMV-4/duck/Delaware/549227/2010 was isolated from oropharyngeal and cloacal swab samples from a hunter-harvested American black duck (*Anas rubripes*) in Delaware in 2010. The complete genome sequence of this isolate was determined by reverse transcription (RT)-PCR using overlapped consensus primers and direct sequencing. The 3′ and 5′ termini were determined by rapid amplification of cDNA ends (RACE) (5). The genome of APMV-4/duck/Delaware/549227/2010 is 15,048 nucleotides (nt) in length. The genomes of APMV-4 strains for which complete sequences have been determined, from Hong Kong (prototype), Korea, Belgium, and South Africa, are each 15,054 nt long (6–9). The Delaware isolate differs in genome length from other reported strains in that it contains a 6-nt-shorter intergenic sequence (IGS) between the fusion (F) and hemagglutinin-neuraminidase (HN) genes. This length polymorphism also was not observed in the partial sequences of five APMV-4 strains isolated from Italy in 2006 (8). The APMV genomes follow the “rule of six,” a requirement that places a constraint on length variation.

The nucleotide sequence identities between the Delaware strain and the Hong Kong, Korean, Belgian, and South African strains are 86.8%, 85.3%, 85.5%, and 85.2%, respectively. The amino acid sequence identities of the nucleocapsid (N), phosphoprotein (P), matrix (M), F, HN, and large (L) proteins between the Delaware strain and the prototype Hong Kong strain are 99.1%, 88.0%, 97.8%, 96.5%, 97.9%, and 96.3%, respectively (8).

The fusion protein of the Delaware strain has a cleavage site sequence of DIQPR↓F, which conforms to previously reported strains from around the world. The HN protein of strain Delaware has a length of 569 amino acids (aa), which is identical to HN protein lengths in strains from Korea and Hong Kong; the HN proteins of the Belgian and Italian strains are 565 aa long, and that of the South African strain is 595 aa long (6–9).

Phylogenetic analysis revealed that the Delaware strain formed a distant separate cluster, demonstrating the genetic diversity of APMV-4. Thus, whereas strain Delaware has 85.2 to 86.8% nucleotide identity to other APMV-4 strains, as noted above, the other strains form a more closely related group; for example, the Hong Kong strain has 90.9 to 91.9% nucleotide identity to other APMV-4 strains. Prior to this study, strain Hong Kong was the most distinct APMV-4 strain, and thus the other strains are even more closely related. This indicates that strain Delaware is genetically the most distant from all other known APMV-4 isolates and represents a new genotype.

### Nucleotide sequence accession number.

The genome sequence of the avian paramyxovirus type 4 strain Delaware has been deposited in GenBank under accession no. JX987283.
